# Polyphenol-Rich Extracts from *Cotoneaster* Leaves Inhibit Pro-Inflammatory Enzymes and Protect Human Plasma Components against Oxidative Stress In Vitro

**DOI:** 10.3390/molecules23102472

**Published:** 2018-09-26

**Authors:** Agnieszka Kicel, Joanna Kolodziejczyk-Czepas, Aleksandra Owczarek, Anna Marchelak, Malgorzata Sopinska, Pawel Ciszewski, Pawel Nowak, Monika A. Olszewska

**Affiliations:** 1 Department of Pharmacognosy, Faculty of Pharmacy, Medical University of Lodz, 90-151 Lodz, Poland; aleksandra.owczarek@umed.lodz.pl (A.O.); anna.marchelak@umed.lodz.pl (A.M.); malgorzata.sopinska@stud.umed.lodz.pl (M.S.); pawel.ciszewski@stud.umed.lodz.pl (P.C.); monika.olszewska@umed.lodz.pl (M.A.O.); 2 Department of General Biochemistry, Faculty of Biology and Environmental Protection, University of Lodz, 90-236 Lodz, Poland; joanna.kolodziejczyk@biol.uni.lodz.pl (J.K-C.); pawel.nowak@biol.uni.lodz.pl (P.N.)

**Keywords:** *Cotoneaster*, oxidative stress, LC-MS, antioxidants, human plasma, peroxynitrite, lipoxygenase, hyaluronidase

## Abstract

The present study investigated the phenolic profile and biological activity of dry extracts from leaves of *C. bullatus*, *C. zabelii* and *C. integerrimus*—traditional medicinal and dietary plants—and evaluated their potential in adjunctive therapy of cardiovascular diseases. Complementary UHPLC-PDA-ESI-MS^3^, HPLC-PDA-fingerprint, Folin-Ciocalteu, and *n*-butanol/HCl assays of the extracts derived by fractionated extraction confirmed that they are rich in structurally diverse polyphenols (47 analytes, content up to 650.8 mg GAE/g dw) with proanthocyanidins (83.3–358.2 mg CYE/g) dominating in *C. bullatus* and *C. zabelii,* and flavonoids (53.4–147.8 mg/g) in *C. integerrimus*. In chemical in vitro tests of pro-inflammatory enzymes (lipoxygenase, hyaluronidase) inhibition and antioxidant activity (DPPH, FRAP), the extracts effects were dose-, phenolic- and extraction solvent-dependent. The most promising polyphenolic extracts were demonstrated to be effective antioxidants in a biological model of human blood plasma—at in vivo-relevant levels (1–5 µg/mL) they normalized/enhanced the non-enzymatic antioxidant capacity of plasma and effectively prevented peroxynitrite-induced oxidative/nitrative damage of plasma proteins and lipids. As demonstrated in cytotoxicity tests, the extracts were safe—they did not affect viability of human peripheral blood mononuclear cells. In conclusion, *Cotoneaster* leaves may be useful in development of natural-based products, supporting the treatment of oxidative stress/inflammation-related chronic diseases, including cardiovascular disorders.

## 1. Introduction

The limitations of endogenous antioxidant system in defense against reactive oxygen/nitrogen species (ROS/RNS) lead to the phenomenon of oxidative stress. The excessive ROS/RNS, formed in these conditions, damage lipids, proteins and DNA, resulting in serious cellular injury, but also initiate intracellular signaling pathways inducing the pro-inflammatory gene expression. In response, the activated inflammatory cells secrete additional reactive species causing further propagation of harmful oxidation processes. This close link between oxidative stress and inflammation is implicated in many chronic conditions including cardiovascular diseases (CVD) -a major cause of mortality in industrialized countries [1,2]. Some alleviation of this civilizational threat may be achieved through consumption of a diet abundant in polyphenol-rich plant materials. Numerous epidemiological studies suggest that regular consumption of foods such as fruits, vegetables, cocoa, tea and wine, distinguished by high content of phenolics, may exert cardio-protective effects in humans. Dietary antioxidants may influence lipid metabolism, inhibit low-density lipoprotein (LDL) oxidation, reduce atherosclerotic lesion formation, inhibit platelet aggregation, as well as improve endothelial function and reduce blood pressure [3,4]. Moreover, the plant-derived polyphenols can increase the total antioxidant capacity of blood plasma, and thus the tolerance of human tissues against ischemic/reperfusion injuries [4]. As the typical diet of Western societies is poor in plant antioxidants, the idea of polyphenol supplementation both for the prevention and supporting therapy of oxidative stress-related ailments gains increasing support. Accordingly, there is a growing interest in finding plant extracts, which may be used for this purpose.

Among numerous plants under study, *Cotoneaster* species appear to be a promising target for closer investigation, as their leaves and fruits are used in traditional Asian medicine as cardiotonic, hypotensive, diuretic and antispasmodic drugs in the treatment of various cardiovascular complaints [5,6]. Additionally, they are also valued as expectorant, antiviral and anticancer agents, and indicated for diabetes mellitus, nasal hemorrhage, excessive menstruation, fever and cough [5,6,7,8]. Furthermore, the fruits and leaves are widely used by Asian communities for culinary purposes [9]. Therapeutic effects of the *Cotoneaster* plant materials are usually linked to the content of phenolic constituents, such as quinic acid pseudodepsides, flavonol glycosides and proanthocyanidins [5,6,9,10]. In our previous study, we investigated phenolic profile and antioxidant activity of the leaves of twelve *Cotoneaster* species cultivated in Poland and selected three species with the highest potential in the context of cardiovascular prevention [10]. Among these, *C. bullatus* and *C. zabelii* exhibited the highest total content of polyphenols with especially large proportion of mono-, di- and trimeric flavan-3-ol derivatives, and *C. integerrimus* was distinguished by the highest level of flavonoids. Both groups of specialized plant metabolites exhibit a wide-range of biological activities, i.e., antioxidant, vaso- and cardioprotective, lipid-lowering, anti-inflammatory, and vasodilatory effects, and may constitute a basis for application of *Cotoneaster* leaves as preventive agents in CVD.

The aim of the present study was a more detailed inquiry into the composition and biological activity of the extracts from leaves of the three most promising species—*C. zabelii*, *C. bullatus* and *C. integerrimus*—as sources of bioactive polyphenols supporting the treatment of oxidative/nitrative stress-induced dysfunctions of the cardiovascular system. The polyphenolic profile of dry extracts prepared by fractionated extraction was investigated by spectrophotometric, UHPLC-PDA-ESI-MS^3^ and HPLC-PDA-fingerprint methods. The biological activity of the extracts was studied in vitro by nine complementary tests in both chemical and human plasma models, covering some of the main molecular mechanisms of anti-inflammatory and antioxidant effects, including inhibition of pro-inflammatory enzymes, free radical scavenging ability, protection of blood plasma components (proteins and lipids) against oxidative/nitrative damage and influence on total non-enzymatic antioxidant capacity of plasma. The relation between the composition and activity was studied using correlation and multiple regression methods. Furthermore, the cellular safety of the extracts towards human peripheral blood mononuclear cells (PBMCs) was preliminary assayed in vitro.

## 2. Results

### 2.1. Phytochemical Standardization of Cotoneaster Leaf Extracts

**Figure 1:**
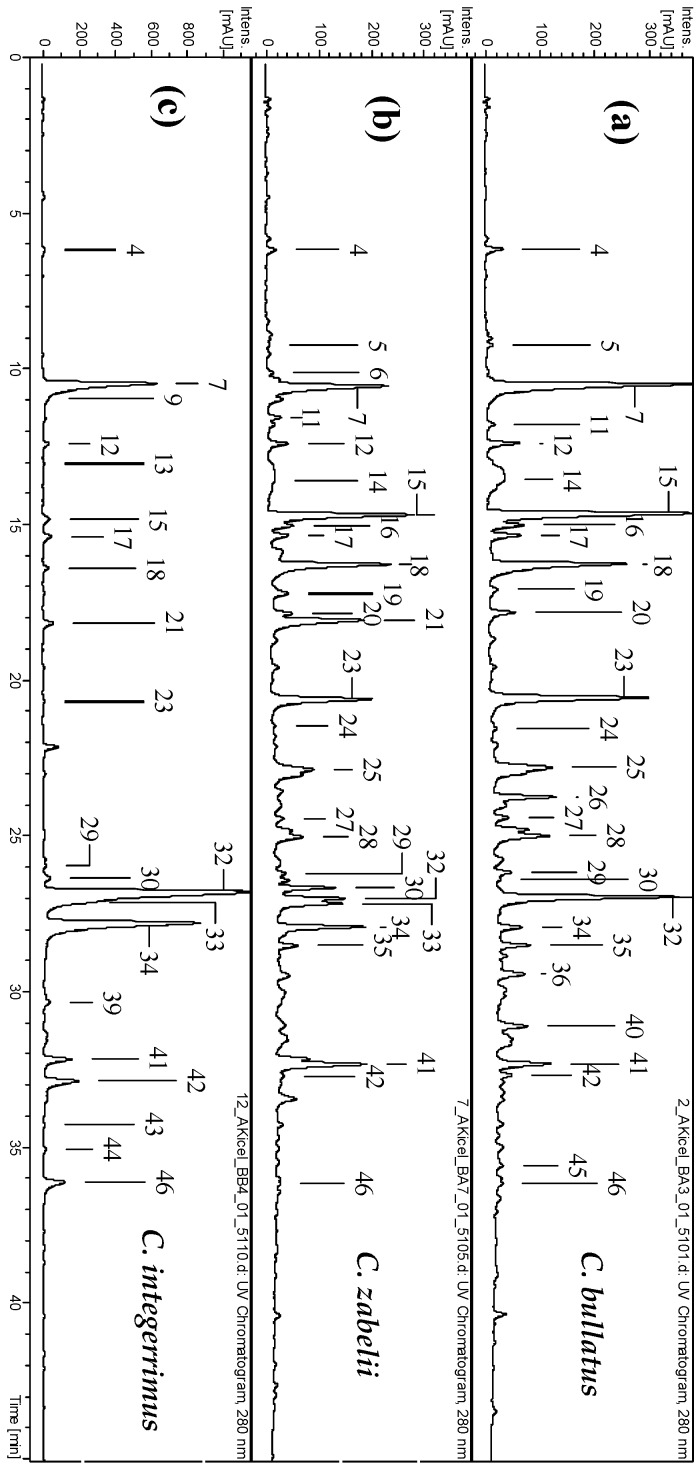
Representative UHPLC-UV chromatograms of the *Cotoneaster* leaf dry extracts at 280 nm; EAF, the ethyl acetate fractions of *C. bullatus* (**a**), *C. zabelii* (**b**) and *C. integerrimus* (**c**). The peak numbers refer to those applied in Table S1.

In the present study, the UHPLC-PDA-ESI-MS^3^ method was used to characterize the composition of the polyphenolic fractions of *C. zabelii*, *C. bullatus* and *C. integerrimus* leaves. In total, over forty phenolic compounds were detected (UHPLC peaks **1**–**47**, Figure 1 and Figure S1, Table S1), 45 of which were fully or partially identified in the leaf dry extracts based on the spectral profiles (UV-Vis and MS/MS), chromatographic behavior, spiking experiments with commercial standards, and literature background [11,12,13]—for identification details see the Supplementary Materials. According to the identification data, three major groups of phenolics could be distinguished, in particular, phenolic acids and related quinic acid pseudodepsides (**3**, **4**, **6**, **7**, **9**, **12**, **13**, **17**, **21**, **42** and **46**), flavonoids (**26**, **30**–**34**, **38**, **39 38**–**41**, **43**–**45** and **47**), and flavan-3-ol derivatives including procyanidins (**5**, **8**, **11**, **14**–**16**, **18**–**20**, **22**–**25**, **27**, **28**, **35** and **36**). The dominating constituents included 5-*O*-caffeoylquinic acid (**7**, chlorogenic acid), (−)-epicatechin (**18**), procyanidin B2 (**15**), procyanidin C1 (**23**), hyperoside (**32**) and isoquercitrin (**34**).

**Figure 2:**
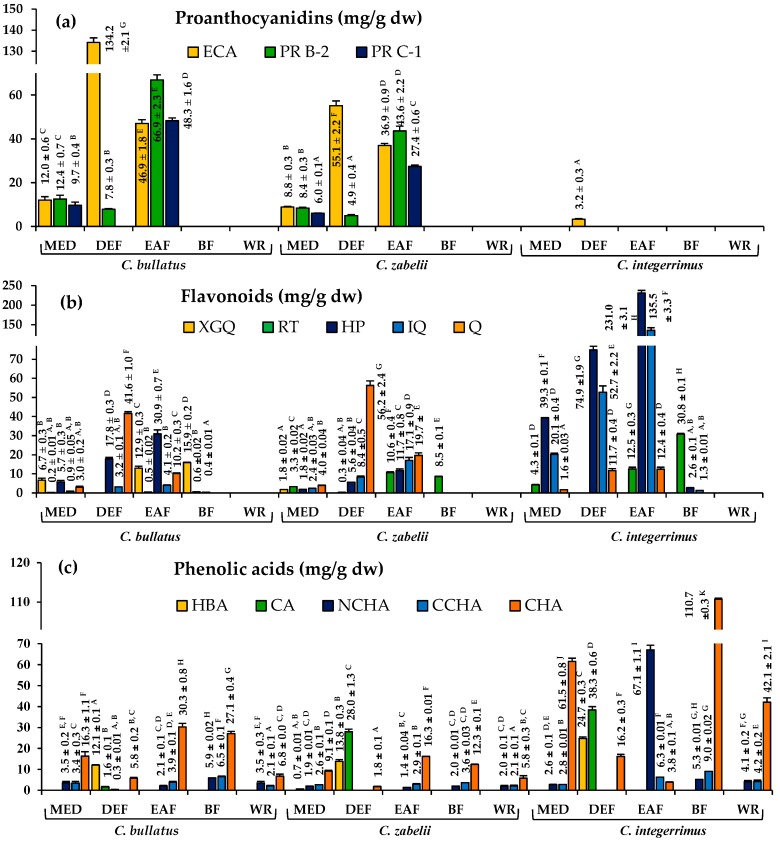
Quantitative profiles of *Cotoneaster* leaf extracts determined by HPLC-PDA: (**a**) the content of individual proanthocyanidins (mg/g dw of the extracts): ECA, (−)-epicatechin; PR-B2, PR-C1, procyanidins B-2 and C-1, respectively; (**b**) the content of flavonoids (mg/g dw): XGQ, quercetin 3-(2″-xylosyl)-galactoside; RT, rutin; HP, hyperoside; IQ, isoquercitrin; Q, quercitrin; (**c**) the content of phenolic acids (mg/g dw): HBA, the sum of hydroxybenzoic acids; CA, caffeic acid; NCHA, 3-*O*-caffeoylquinic acid; CCHA, 4-*O*-caffeoylquinic acid; CHA, 5-*O*-caffeoylquinic acid (chlorogenic acid), respectively. Extracts: MED, methanol-water (7:3, *v*/*v*) extract; DEF, diethyl-ether fraction; EAF, ethyl acetate fraction; BF, *n*-butanol fraction; WR, water residue. Data expressed as mean values ± SD (*n* = 3). For each analyte, different superscript capitals indicate significant differences (*p* < 0.05).

According to the LC-MS results, the total contents of polyphenols (TPC), proanthocyanidins (TPA), flavonoids (TFC) and phenolic acids (TAC) were selected as standardization targets of the extracts. As presented in Table 1, the TPC level in the MED extracts, determined by the standard Folin-Ciocalteu method, varied in the range of 241.3–332.9 mg GAE/g dw of the extract, depending on the studied species, with the highest values found for *C. bullatus* and *C. zabelii.* The fractionation of the MEDs between solvents of different polarity led to a further enrichment in polyphenols, with the highest TPC level obtained for EAFs (470.9–650.8 mg GAE/g dw) and DEFs (453.1–546.9 mg GAE/g dw). The level of phenolics in the examined fractions, verified by HPLC-PDA as a sum of individual phenolics and expressed in the equivalents of authentic standards, was in the range of 12.4–505.7 mg/g dw, with the peak values still obtained for EAFs (244.7–505.7 mg/g dw) and DEFs (286.5–375.9 mg/g dw). In the fractions of *C. bullatus* and *C. zabelii*, the most abundant phenolic group were proanthocyanidins, while in the case of the *C. integerrimus* fractions, flavonoids and phenolic acids were the dominant compounds (Table 1, Figure 2).

The total content of proanthocyanidins in the *Cotoneaster* dry extracts varied over a relatively wide range, constituting up to 80–86% of their TPC values, depending on the assay applied (Table 1). In the photometric butanol/HCl test, the TPA content expressed as cyanidin chloride equivalents (CYE) was significantly higher (9.7–434.9 mg CYE/g dw) than TLPA values obtained for the corresponding fractions using HPLC-PDA and understood as the sum of individual proanthocyanidins, calculated as (−)-epicatechin equivalents (9.1–278.7 mg/g dw). Moreover, the results of photometric assay indicated EAF and BF of *C. bullatus* and *C. zabelii* as the most abundant in proanthocyanidins, while based on HPLC-PDA results, the DEF and EAF of the same species contained the highest amount of these compounds. This discrepancy might be connected with the profile of proanthocyanidin fraction (the monomers to oligomers ratio) and specific reactivity of individual molecules in particular tests [14]. The *n*-butanol/HCl assay gives positive responses for analytes varying from monomers—but excluding (−)-epicatechin—to highly polymerized molecules. On the other hand, the RP-HPLC method enables only separation and detection of low- and medium-molecular weight proanthocyanidins (up to tetramers), including (−)-epicatechin and procyanidins B-2 and C-1, which dominated among the constituents of the less hydrophilic fractions, i.e., DEFs and EAFs (Figure 2).

The level of flavonoids, determined by HPLC-PDA as a sum of individual glycosides, was in the range of 12.7–403.5 mg/g dw, with the highest values found for EAF and DEF of *C. integerrimus* in which the TFC constituted 85.7 and 31.9% of their TPC levels, respectively (Table 1). As regard of individual flavonoids, hyperoside and isoquercitrin were the dominant compounds and constituted up to 53% of the TFC level of these fractions (Figure 2). In all of the other extracts, the flavonoid content was at least five-fold lower (12.7–81.8 mg/g dw) and constituted at most 16.3% of the TPC value.

The content of phenolic acids in the investigated *Cotoneaster* extracts considerably varied, ranging between 12.4 and 147.8 mg/g dw (Table 1), with the highest value observed for DEF of *C. integerrimus*, for which phenolic acids were the dominant constituents (32.6% of TPC). In general, DEFs accumulated primarily simple hydroxybenzoic and hydroxycinnamic acids (protocatechuic, *p*-hydroxybenzoic and caffeic acids), while in all other extracts monocaffeoylquinic acid isomers dominated, with the highest content of chlorogenic acid (110.7 mg/g dw) observed in BF of *C. integerrimus* (Table 2).

### 2.2. Antioxidant Activity of Leaf Extracts in Chemical Models

To preliminarily evaluate the antioxidant activity of the investigated extracts we applied two of the most frequently used tests (DPPH and FRAP). The results are reported in Table 2. Regardless of the *Cotoneaster* species tested, all extracts showed significant and dose-dependent activity with a wide range of final capacities, depending on the extraction solvent. Furthermore, the results obtained in the DPPH (EC_50_ = 3.2–27.9 µg/mL, TEAA = 0.8–6.9 mmol TX/g) and FRAP tests (FRAP = 3.8–17.7 mmol Fe^2+^/g dw, TERP = 1.2–6.9 mmol TX/g dw) were highly consistent, which is reflected in a significant linear correlation (*r* = −0.8841, *p* < 0.001). As far as phenolic levels are concerned, the strong relationships in correlation studies were observed between the activity parameters and the TPC levels (*r* > 0.96, *p* < 0.001, Table S2) as well as TLPA levels (*r* > 0.66, *p* < 0.01, Table S2). On the other hand, the multiple regression models indicated a significant influence of all of the investigated groups of compounds (Table S3). It is also noticeable that, in the case of both antioxidant methods, correlations with the TPC levels were even stronger than those found previously for the methanol-water (7:3, *v*/*v*) liquid extracts [10], which could be a consequence of the purification and concentration of phenolics during the preparation of dry extracts. The fractionation of the crude MED extracts between solvents of different polarity yielded the fractions with various activity, and, depending on the *Cotoneaster* species and the test used, the EAF, DEF or BF were indicated as the most active. Among them, the antioxidant capacity of EAFs of *C. bullatus* and *C. zabelii* was higher or not statistically different (*p* < 0.05) than that of BHA or Trolox^®^ (6-hydroxy-2,5,7,8-tetramethylchroman-2-carboxylic acid), a synthetic analog of vitamin E.

### 2.3. Antioxidant Activity of Leaf Extracts in Human Plasma Model

**Figure 3:**
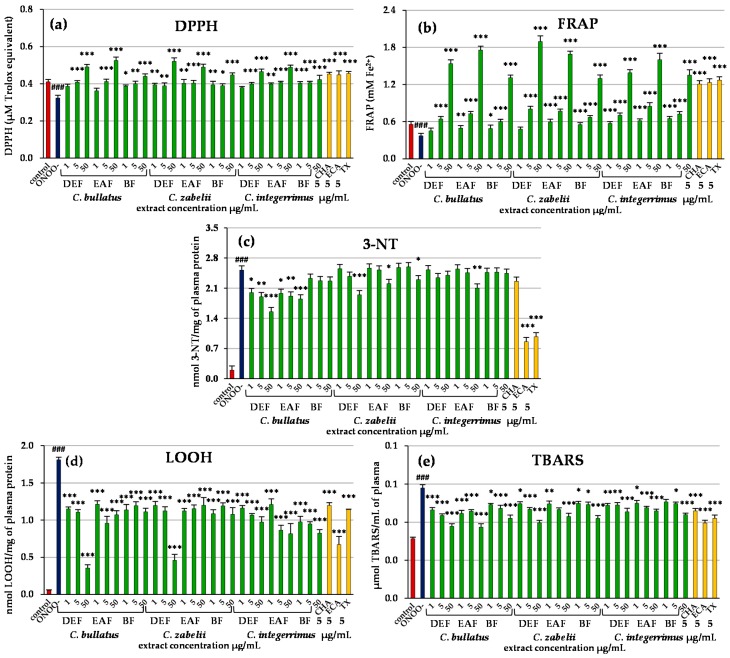
Effects of *Cotoneaster* leaf extracts on human plasma exposed to oxidative stress: (**a**) free radical scavenging ability, DPPH; (**b**) influence on ferric reducing ability of plasma, FRAP; (**c**) effects on the nitration of tyrosine residues in plasma proteins and formation of 3-nitrotyrosine, 3-NT; (**d**,**e**) effects on the peroxidation of plasma lipids including (**d**), formation of lipid hydroperoxides, LOOH and (**e**), formation of thiobarbituric acid-reactive substances, TBARS. Results are expressed as means ± SE (*n* = 5) for repeated measures: ### *p* < 0.001, for ONOO^−^-treated plasma (without the extracts) versus control (untreated) plasma, and * *p* < 0.05, ** *p* < 0.001, *** *p* < 0.001, for plasma treated with ONOO^−^ in the presence of the investigated extracts (1–50 µg/mL) or the standards (5 µg/mL). Extracts: DEF, diethyl-ether fraction; EAF, ethyl acetate fraction; BF, *n*-butanol fraction. Standards: CHA, chlorogenic acid; ECA, (−)-epicatechin; TX, Trolox^®^.

The DEF, EAF and BF extracts were evaluated further in an experimental model of human blood plasma under oxidative stress conditions induced by peroxynitrite (ONOO¯). In the absence of the extracts, the exposure of the plasma to 100 µM of ONOO^−^ resulted in a decrease of its non-enzymatic antioxidant capacity as observed in the DPPH and FRAP assays (Figure 3a,b). This effect was normalized by the extracts—in the majority of cases even at the lowest concentration of 1 µg/mL (Figure 3a,b, *p* > 0.05 vs. the control plasma). The most significant improvement in the plasma reducing and free radical scavenging ability was observed for DEF of *C. zabelii* and EAFs of *C. bullatus* and *C. integerrimus*, with the strongest effects recorded for their highest concentrations (50 µg/mL). For example, in the plasma samples pre-incubated with 50 µg/mL of the *C. zabelii* DEF, the FRAP parameter increased by about 411% (Figure 3b), while the DPPH-scavenging capacity by about 61% (Figure 3a), in comparison to the ONOO^−^-treated plasma. In general, the antioxidant capacities of the extracts, especially those recorded in the FRAP assay, were dose-dependent.

The exposure of the plasma to 100 µM ONOO^−^ resulted also in oxidative/nitrative alterations of plasma proteins and lipids, as demonstrated by the increase (*p* < 0.001, vs. the control plasma) in the levels of oxidative stress biomarkers: 3-nitrotyrosine (3-NT, Figure 3c), lipid hydroperoxides (LOOH, Figure 3d) and thiobarbituric acid-reactive substances (TBARS, Figure 3e). As shown in Figure 3c–e, the extent of this damage was significantly reduced in the presence of the extracts. For instance, the DEF and EAF of *C. bullatus* (1–50 µg/mL) as well as DEF of *C. zabelii* (50 µg/mL) effectively diminished the nitration of tyrosine residues caused by ONOO^−^, and we could observe up to 36% reduction in 3-NT level in the plasma (Figure 3c, *p* < 0.001). As the analysis of the lipid peroxidation biomarkers (LOOH and TBARS) demonstrated, the leaf extracts were also able to protect the plasma lipids, and this effect was noticeable and statistically significant for the whole range of concentrations (1–50 µg/mL). For example, in the LOOH test, EAF and BF of *C. bullatus* and *C. zabelii* reduced the peroxidation parameters by up to 47% (Figure 3d). The strongest activity was observed for DEFs of these two species at 50 µg/mL—the LOOH level decreased by 80 and 79%, respectively. In the case of *C. integerrimus*, the EAF and BF displayed the strongest effects (1–50 µg/mL, 33–55% inhibition of LOOH formation). In the TBARS test all leaf extracts revealed similar antioxidant ability, reducing peroxidation parameters within a narrow, dose-dependent range of 26–30% (Figure 3e).

In all tests, except for 3-NT formation, the activity of the extracts (at least at the highest concentration) was comparable to or higher than that of positive standard (Trolox^®^). The correlation studies for all methods, apart from the LOOH assay, showed significant or close to significant relationships with the TPC and TLPA values (Table S2), and multiple regression models indicated TLPA as the main determinant of the investigated activities (Table S3). The influence of polyphenols was further confirmed by the significant activity of the two phenolic standards representing the main groups of *Cotoneaster* constituents, i.e., chlorogenic acid and (−)-epicatechin, with the latter especially efficient in the 3-NT test (Figure 3a–e).

### 2.4. Inhibitory Effect of Leaf Extracts on Pro-Inflammatory Enzymes

To examine the potential anti-inflammatory effect of the studied *Cotoneaster* extracts, inhibition of lipoxygenase (LOX) and hyaluronidase (HYAL) activity was tested. As presented in Table 3, the extracts reduced activity of both enzymes in a concentration-dependent manner. Considering the IC_50_ values converted to µg/U of enzyme, the inhibitory effectiveness of the extracts was similar toward both enzymes with the exception of EAFs, which were stronger inhibitors of LOX than HYAL.

The most active extract was BF of *C. bullatus*, the effects of which were higher or not statistically different than those of positive controls including indomethacin, an anti-inflammatory drug (Table 3). In the case of the LOX inhibition, the correlation studies showed a strong relationship between the activity parameters and the TPC levels (*r* = −0.8403, *p* < 0.001, Table S2) with a significant impact of the TAC, TPA and TLPA fractions revealed by the multiple regression models (Table S3). On the contrary, no statistical relationships were found for the HYAL assay.

### 2.5. Influence of the Leaf Extracts on Cell Viability

**Figure 4:**
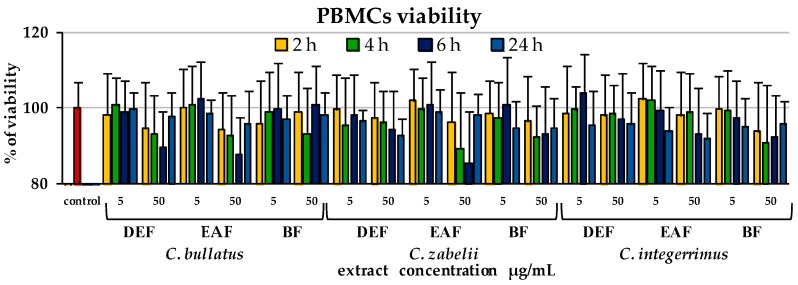
Viability of peripheral blood mononuclear cells (PBMCs) after 2, 4, 6 and 24 h incubation with the *Cotoneaster* leaf extracts at 5 and 50 µg/mL. Results are presented as means ± SD (*n* = 10). Extracts: DEF, diethyl-ether fraction; EAF, ethyl acetate fraction; BF, *n*-butanol fraction.

The cellular safety of the *Cotoneaster* extracts (at the concentration levels of 5 and 50 µg/mL) was evaluated in the model of PBMCs using Trypan blue as a diagnostic dye, after 2, 4, 6 and 24 h of incubation. Regardless of the extract concentration and the incubation time, the viability of the extract-treated cells did not differ significantly from that of the control, untreated cells (Figure 4, *p* > 0.05) and remained at high level constituting 91.0–102.3% of the control viability. In consequence, no cytotoxic effects were found.

## 3. Discussion

The present study is the continuation of our research exploring the potential of *Cotoneaster* leaves in the prevention and treatment of oxidative stress-related pathologies, in particular those related to the cardiovascular system. In our previous work we preliminarily evaluated twelve *Cotoneaster* species cultivated in Poland and demonstrated that the leaves of the plants are good sources of polyphenols with corresponding antioxidant activity [10]. Most of the investigated leaves were characterized by the domination of flavan-3-ol derivatives in their phenolic profile, and among those, the leaves of *C. bullatus* and *C. zabelii* were distinguished by the highest content of both flavan-3-ols and total polyphenols as well as the best activity parameters. On the other hand, the leaves of *C. integerrimus* contained especially high amounts of flavonoids. According to the known beneficial effects of flavan-3-ols and flavonoids within the cardiovascular system [15,16], these three species were selected for further studies in the present work.

For the purpose of health-supporting application of medicinal or dietary plants, modern phytotherapy recommends the use of standardized dry extracts containing purified and concentrated active compounds with enhanced activity parameters [17]. To account for this fact, in the present work we prepared dry extracts and fractions of the selected *Cotoneaster* leaves using fractionated extraction and solvents of different polarity. The starting point for fractionation were crude methanol-water (7:3, *v*/*v*) extracts (MEDs) prepared with the solvent indicated previously as the most effective extractant of *Cotoneaster* polyphenols [10]. The fractionation step allowed for enrichment of the extracts in selected analytes, e.g., simple phenolic acids, flavonoid aglycones and monomeric flavan-3-ol derivatives were concentrated in DEFs, flavonoid monoglycosides and di-, trimeric flavan-3-ols in EAFs, and flavonoid diglycosides with polymeric procyanidins in BFs (Figure 2). Additionally, the total content of polyphenols in the fractions, especially in DEFs and EAFs, was significantly enhanced in comparison to the crude MEDs, placing them among the most polyphenol-rich products, such as extracts derived from green tea leaves, in a liquid or dried form (450–900 mg/g dw), or commercial extract of chokeberry (700 mg/g) [18,19]. Purification of the fractions enabled also effective identification of eighteen new *Cotoneaster* constituents, undetected previously [10] in the crude extracts due to the low content.

As the antioxidant capacity of natural products is one of the most important aspects of their beneficial effects in CVD [20], our inquiry into the activity of the obtained extracts started with the DPPH and FRAP assays, simple chemical tests of antioxidant potential reflecting direct interactions of the extracts constituents with free-radicals and transition metal ions. The results proved the effectiveness of the extracts in both models with the activity parameters of the most active DEF and EAF fractions comparable to those of Trolox^®^ and BHA (Table 2). Interestingly, the comparison of our results from the DPPH test with the obtained earlier for other *Cotoneaster* representatives suggests that the investigated extracts might be superior to those of the twigs of *C. nummularia* [6] and the leaves of *C. melanocarpus* [7]. Additionally, the TEAA value for the MED of *C. integerrimus* leaves (1.61 mmol Trolox^®^/g dw) observed in the present study was higher than the corresponding value for leaf methanol extract of this species (0.76 mmol Trolox^®^/g) found by Uysal et al. [9], which suggests in turn that the phytochemical and biological value of *Cotoneaster* plants might be significantly influenced by environmental conditions.

As the extracts are complex matrices, their activity may be associated with constituents that are not detectable by the usually used phytochemical assays. Thus, to confirm that the observed activity is in fact polyphenol-related, we performed statistical analyses employing simple and multiple regression models. As a result, a high correlation of the obtained results with the TPC levels (Table S2) was demonstrated, which is not that much conclusive as all three compared assays (TPC, DPPH, FRAP) are based on the same SET (Single Electron Transfer) mechanism. More importantly, the analysis of correlations between the effectiveness of the extracts in the FRAP/DPPH tests and the content of particular groups of compounds (Table S2) indicated low molecular-weight flavan-3-ol derivatives (TLPA) as the main determinants of the tested activity. Additionally, the application of multiple regression (Table S3) allowed for visualization of all of the effects hidden in the data. In the obtained models, the level of flavan-3-ols still remained highly relevant, but the significance of flavonoids and phenolic acids was also highlighted.

For a more detailed insight into the role that the leaf polyphenols might play in in vivo conditions, we evaluated the ability of DEFs, EAFs and BFs (the most active extracts in chemical models) to prevent negative effects of peroxynitrite (ONOO^−^) in the blood plasma. Peroxynitrite is a powerful, in vivo-operating oxidant that damages the structure of lipids, proteins and DNA via direct oxidative reactions or indirect, radical-mediated mechanisms and, therefore, is a potent inducer of cell death [21]. It is synthesized in vivo from NO^•^ and O_2_^−•^, and its production (and thus cytotoxic effects), may be enhanced even up to 50–100 µM per min in case of increased NO^•^ generation, e.g., during inflammation or reperfusion injury [21,22]. The harmful action of ONOO^−^ is reflected in the decrease of the non-enzymatic antioxidant status of plasma (DPPH and FRAP assays) and in the increase in the levels of oxidative stress biomarkers, mainly markers of protein nitration (level of 3-nitrotyrosine) and lipid peroxidation (level of lipid hydroperoxides and TBARS). Accumulated results of clinical studies indicate that the evident changes in these parameters can be observed in patients with various CVD, including atherosclerosis, stroke, myocardial infarction and chronic heart failure, and they correspond to the level of intravascular damage, instability of atherosclerotic plaque, impairment of endothelial function, or pro-inflammatory intravascular effects [23,24]. The lack of specific endogenous enzymes responsible for deactivation of ONOO^−^ has raised a considerable interest in search for the effective antioxidants, which are able to scavenge this molecule or to diminish its harmful effects. As the results of our study demonstrated (Figure 3), the investigated *Cotoneaster* leaf extracts were able to reduce the negative ONOO^−^ impact on plasma components in concentrations as low as 1–5 µg/mL, which corresponds to the phenolics level of 0.1–3.25 µg GAE/mL. It is important from the physiological point of view, as these are the levels that can be achieved in plasma after oral administration of polyphenol rich products. For instance, the maximum concentration of (−)-epicatechin in the plasma of volunteers after ingestion of a 70–140 mg dose of an apple-derived flavanol-rich extract was, in the range of 3.5–8.9 µM, i.e., 1.0–2.6 µg/mL [25]. A single dose of quercetin (500 mg) from different quercetin-fortified food products, resulted in its plasma concentration in the range of 0.5–1.0 µg/mL [26], while an oral ingestion of quercetin mono- and diglycosides (100–200 mg)—contributed to the plasma concentration, ranging between 0.30 and 2.3 µg/mL [27]. In the case of monocaffeoylquinic acid derivatives, a maximal concentration in the range of 0.3–2.1 µg/mL (depending on the isomer) was determined after ingestion of green coffee extract with the total dose of all isomers about 100 mg [28].

The additional high concentration (50 µg/mL) allowed us to observe amplified effects and their statistical analysis (Tables S2 and S3), which in case of the DPPH, FRAP, TBARS and 3-NT tests in plasma provided similar results indicating TLPA level as the main determinant for the activity. This effect was especially evident in the case of protein nitration (as the only two extracts that exhibited statistically significant activity in this assay were also the two with the highest content of low molecular-weight flavan-3-ols), and was further confirmed by particularly high efficacy of (−)-epicatechin in the 3-NT test. Indeed, (−)-epicatechin and/or its oligomers have been reported previously to effectively protect against the toxicity of ONOO^−^ toward blood platelets and plasma [29]. Two possible mechanisms of its activity have been proposed—direct scavenging of ONOO¯ and/or products of its decomposition, especially OH^•^ and NO_2_^•^ radicals, or interference with intermediate products of ONOO^−^ reactions with the target structures, that enables the reversal of the initial damage. (−)-Epicatechin itself has been found to be a potent scavenger of ONOO¯, and an efficient attenuator of the protein nitration, including both tyrosine nitration and tyrosine dimerization [29].

Apart from the impact of TLPA and (−)-epicatechin, the effects of the other groups of compounds were too weak to be detected in the study design applied in the present work, but the results from the chemical tests, and the statistically significant effects demonstrated for chlorogenic acid standard in most of the tests, indicate that they might also have their role to play in the overall extracts capacity.

In the case of the LOOH assay, a direct correlation between the TPA values and the levels of lipid hydroperoxides was found (Tables S2 and S3), which would implicate a counterintuitive negative impact of this group of compounds on the studied antioxidant activity. However, this relationship was mostly a consequence of high activity of *C. zabelii* and *C. bullatus* DEF extracts, which apart from containing low levels of TPA, were also distinguished by high content of quercetin. Indeed, an additional statistical test indicated a strong and this time negative correlation between the quercetin content in the extracts and the LOOH levels in the plasma samples (*r* = − 0.7798, *p* < 0.05), thus allowing for ascribing the activity in the LOOH test to this flavonol aglycone.

Inflammation, closely related to oxidative stress, is also implicated in pathogenesis of many chronic diseases, including CVD. This process involves multiple factors, including immune cells, inflammatory mediators, and a number of enzymes [30]. Lipoxygenases and hyaluronidases are some of the targets proposed for the treatment of inflammatory-related complaints. The lipoxygenases (LOX) catalyze the incorporation of dioxygen molecules into polyunsaturated fatty acids, and lead to the formation of corresponding hydroperoxides, which is a first step in biosynthesis of leukotrienes, pro-inflammatory mediators, the action of which includes coronary artery contraction, chronotropic effect on heart rate, and impairment of left ventricular contractility [31,32]. The second enzyme, hyaluronidase, known as a spreading factor, hydrolyzes hyaluronic acid, an important constituent of, i.a. endothelial surface layer, the disruption of which causes endothelium dysfunction and increases the instability of the atherosclerotic plate [33,34]. As the results of the present study indicated (Table 3), the *Cotoneaster* extracts, rich in polyphenols, were found to be efficient inhibitors of both tested enzymes, with the most active BF and DEF extracts, the effects of which were comparable with that of indomethacin, a non-steroidal anti-inflammatory agent. The LOX inhibition was highly polyphenol-related (Tables S2 and S3), and the multiple regression model indicated a relevant influence of flavan-3-ols and phenolic acids. On the other hand, the activity of the quercetin standard, does not allow to exclude flavonoids as the relevant mediators of the investigated activity. Their role might have been underestimated by the model due to the statistically significant correlation between the content of flavonoids and phenolic acids. The lack of any statistical relationships in the case of the HYAL inhibition (Table S2), might indicate some complex synergistic interactions between the constituents, and further more detailed research is required to fully explain that fact.

The centuries-long traditional use of the *Cotoneaster* species is a confirmation of their safety. However, the potential application of the extracts in the concentrated form requires a closer investigation into their possible harmful effects. In this context, the lack of cytotoxicity of the extracts towards the PBMCs (Figure 4) constitutes an additional evidence of their value.

## 4. Materials and Methods

### 4.1. General Experimental Procedures

HPLC or analytical grade reagents and standards such as 2,2-diphenyl-1-picrylhydrazyl (DPPH), 2,4,6-tris-(2-pyridyl)-s-triazine (TPTZ), bovine testis hyaluronidase, lipoxygenase from soybean, *o*-phenylenediamine dihydrochloride (SigmaFast™ OPD, Merck KGaA, Darmstadt, Germany), 2-thiobarbituric acid, Histopaque^®^-1077 medium (Merck KGaA, Darmstadt, Germany), (Ca^2+^ and Mg^2+^)-free phosphate buffered saline (PBS); (±)-6-hydroxy-2,2,7,8-tetramethylchroman-2-carboxylic acid (Trolox^®^), butylated hydroxyanisole (BHA), gallic acid monohydrate (GA), quercetin dehydrate, chlorogenic acid hemihydrate (5-*O*-caffeoylquinic acid), 3-*O*- and 4-*O*-caffeoylquinic acids, *p*-hydroxybenzoic acid, caffeic acid, protocatechiuc acid, hyperoside semihydrate, isoquercitrin, and rutin trihydrate were obtained from Sigma-Aldrich (St. Louis, MO, USA), while procyanidins B-2 and C-1, and (−)-epicatechin were purchased from PhytoLab GmbH (Vestenbergsgreuth, Germany). The standards of quercitrin (quercetin 3-*O*-α-rhamnoside) and quercetin 3-*O*-β-(2″-*O*-β-xylosyl)-galactoside have been previously isolated in our laboratory from *C. bullatus* and *C. zabelii* leaves with at least 95% HPLC purity (unpublished results). Peroxynitrite was synthesized according to [35]. Anti-3-nitrotyrosine polyclonal antibody, biotin-conjugated secondary antibody, and Streptavidin/HRP were purchased from ThermoFisher Scientific (Chelmsford, MA, USA) or from Abcam (Cambridge, UK). HPLC grade solvents as acetonitrile and formic acid were from Avantor Performance Materials (Gliwice, Poland).

For chemical tests of antioxidant activity, the samples were incubated at constant temperature using a BD 23 incubator (Binder, Tuttlingen, Germany) and measured using a UV-1601 Rayleigh spectrophotometer (Beijing, China). Enzymes inhibition assays and activity tests in a human plasma model were performed in 96-well plates and monitored using a microplate reader SPECTROStar Nano (BMG Labtech, Ortenberg, Germany). Cellular safety tests were conducted using a microchip-type automatic cell counter BioRad (Hercules, CA, USA). The UHPLC-PDA-ESI-MS^3^ and HPLC-PDA studies were performed using the equipment described previously [10,36].

### 4.2. Plant Material and Preparation of Dry Extracts

Leaf samples of *C. bullatus* Bois, *C. zabelii* C.K. Schneid. and *C. integerrimus* Medik. were collected in September 2013, in the Botanical Garden (BG; 51°45′ N 19°24′ E) in Lodz (Poland) and in the Arboretum (AR; 51°49′ N 19°53′ E), the Forestry Experimental Station of Warsaw University of Life Sciences (SGGW) in Rogow (Poland). The voucher specimens were deposited in the Herbarium of the Department of Pharmacognosy, Medical University of Lodz (Poland) with the following numbers: KFG/13/CBL, KFG/13/CZB and KFG/13/CIN, respectively.

The leaf samples were air-dried under normal conditions, powdered with an electric grinder, and sieved through a 0.315-mm sieve. A portion (60 or 100 g) of the pulverized plant material was first extracted with chloroform (1 L, 48 h, a Soxhlet apparatus) to remove lipids and waxes (the chloroform extracts was discarded), and then refluxed four times with methanol-water (7:3, *v*/*v*) (4 × 1 L × 6 h) to give the defatted methanol extract (MED). The obtained extract was dissolved in water (1 L) and subjected to sequential liquid-liquid extraction (10 × 150 mL each) with diethyl ether, ethyl acetate and *n*-butanol, successively. The three fractions of diethyl ether (DEF), ethyl acetate (EAF) and *n*-butanol (BF), as well as the water residue (WR), prepared independently for each of the *Cotoneaster* species, were evaporated in vacuo and lyophilized using an Alpha 1–2/LD Plus freeze dryer (Christ, Osterode am Harz, Germany). The MED, DEF, EAF and BF extracts were evaluated according to standard TLC procedures [37], and none of lipid compounds were found (unpublished results).

### 4.3. Phytochemical Profiling

The total phenolic content (TPC) in the test extracts was quantified according to the Folin-Ciocalteu method as described previously [38]. The results were expressed as gallic acid equivalents (GAE) per g of dry weight of the extracts (mg GAE/g dw). The total proanthocyanidin content (TPA) in the extracts was determined on the base of the modified *n*-butanol-HCl assay as described previously [39]. The results were expressed as cyanidin chloride equivalents (CyE) per g of dry weight of the extracts (mg CyE/g dw).

Qualitative and quantitative studies of individual phenolic compounds were performed by UHPLC-PDA-ESI-MS^3^ and HPLC-PDA analyses using the same procedures as described previously ([36], [10] respectively]). In qualitative studies, eleven external standards were used for calibration, i.e., protocatechuic, *p*-hydroxybenzoic, chlorogenic and caffeic acids, (−)-epicatechin, procyanidin B-2, rutin, hyperoside, isoquercitrin, quercitrin, and quercetin 3-*O*-β-(2″-*O*-β-xylosyl)-galactoside. Peak identification was made by comparison of retention times, UV-Vis and MS spectra with the reference compounds. The tentatively identified peaks, depending on their PDA spectra, were quantified as equivalents of (−)-epicatechin (proanthocyanidins), *p*-hydroxybenzoic acid (hydroxybenzoic acids), chlorogenic acid (chlorogenic acid isomers), caffeic acid (other hydroxycinnamic acid derivatives), rutin (flavonoid diglycosides) and hyperoside (flavonoid monoglycosides).

### 4.4. Antioxidant Activity in Chemical Models

The DPPH scavenging activity was determined according to the earlier optimized method [40] using serial dilutions of the extracts (1.3–33.4 µg/mL) in methanol-water (7:3, *v*/*v*). The EC_50_ values (μg/mL) were calculated from five-point concentration-inhibition curves, and converted to Trolox^®^ Equivalent Antioxidant Activity (TEAA, mmol TX/g dw). The standards of QU, ECA, CHA, BHA and TX were used as positive controls. The FRAP (Ferric Reducing Antioxidant Power) was assayed as described previously [38] and expressed in mmol of ferrous ions (Fe^2+^), produced by 1 g of the extracts or standards (the same positive controls as used in the DPPH tests) as calculated from the calibration curve of ferrous sulphate, and in the Trolox^®^ Equivalents Reducing Power (TERP, mmol TX/g dw).

### 4.5. Antioxidant Activity in Human Plasma Models

#### 4.5.1. Isolation of Blood Plasma and Sample Preparation

Human blood from healthy volunteers came from the Regional Centre of Blood Donation and Blood Treatment in Lodz (Poland). All volunteers gave their informed consent for inclusion before they participated in the study. The study was conducted in accordance with the Declaration of Helsinki, and the protocol was approved by the Ethics Committee at the Medical University of Lodz RNN/347/17/KE. The plasma samples were obtained by centrifugation, performed according to [41]. Plasma samples, diluted then with 0.01 M Tris/HCl pH 7.4 (1:4 *v*/*v*) were pre-incubated for 15 min at 37 °C with the examined extracts, added to the final concentration range of 1–50 µg/mL, and then exposed to 100 or 150 µM peroxynitrite (ONOO^−^). Control samples were prepared with plasma untreated with the extracts and/or peroxynitrite. To eliminate the possibility of direct interactions of the extracts with plasma proteins and lipids, control experiments with blood plasma and the extracts only (without adding ONOO^−^) were also performed and no pro-oxidative effect was found.

#### 4.5.2. Evaluation of Oxidative and Nitrative Damage to Blood Plasma Proteins and Lipids

The 3-nitrotyrosine-containing proteins were detected by the competitive ELISA test (enzyme-linked immunosorbent assay) as described previously [42]. The concentrations of nitrated proteins that inhibit anti-nitrotyrosine antibody binding were estimated from the standard curve of 3-nitrofibrinogen (3-NT) and expressed as the 3NT-Fg equivalents (in nmol/mg of plasma protein). The formation of lipid hydroperoxides in plasma samples was assessed with the use of ferric-xylenol orange (FOX-1) protocol [42]. The amount of lipid hydroperoxides (LOOH) was calculated from the standard curve of hydrogen peroxide and expressed in nmol LOOH/mg of plasma proteins. The determination of the thiobarbituric acid-reactive substances (TBARS) was conducted according to the previously described method [42]. The TBARS concentration was expressed in μmol TBARS/mL of plasma.

#### 4.5.3. Determination of the Non-Enzymatic Antioxidant Capacity of Blood Plasma (NEAC)

The influence of the extracts on the NEAC of blood plasma was assessed by the DPPH and FRAP tests according to the methods described previously ([42,43] respectively). The DPPH scavenging activity of plasma in the presence of the extracts was expressed in micromolar Trolox^®^ equivalents in plasma (µM TX), while the FRAP reactivity was expressed in mM Fe^2+^ in plasma.

#### 4.5.4. Inhibition of Pro-Inflammatory Enzymes

The ability of the leaf extracts to inhibit lipoxygenase (LOX) and hyaluronidase (HYAL) was determined on the base of the earlier optimized methods [42]. The results of both tests were expressed as IC_50_ values (µg/mL), calculated from concentration-inhibition curves, and converted to µg/U of enzyme.

#### 4.5.5. Cellular Safety Testing

The potential cytotoxicity of the examined extracts was evaluated using an experimental system of peripheral blood mononuclear cells (PBMCs) according to [44]. PBMCs were isolated from fresh human blood using the Histopaque^®^-1077 medium (Merck KGaA, Darmstadt, Germany), according to the protocol provided by the manufacturer and suspended in 0.02 M PBS to obtain a cell count of 1 × 10^6^ PBMCs/mL. The cell samples were incubated with the leaf extracts (the final concentrations of 5 and 50 µg/mL) at 37 °C for 2, 4, 6 and 24 h of incubation. PBMCs viability was estimated in a routine dye excluding test, based on a staining with 0.4% Trypan blue [45]. The procedure was carried out according to the manufacturer’s protocol. Additionally, the isolated PBMCs were suspended in the RPMI-1640 medium (3 × 10^6^ PBMCs/mL) and incubated with the extracts (5 and 50 µg/mL) for 24 h (in 96-well microplates, at 37 °C, in a humidified atmosphere, with 5% CO_2_). The measurements were performed as described above.

### 4.6. Statistical Analysis

The results were reported as means ± SD (standard deviation) or ± SE (standard error) for the indicated number of experiments. Normality of the distribution of the results was verified using the Shapiro-Wilk test, and the homogeneity of variances using the Levene’s test. The significance of differences between samples and controls was determined with one-way ANOVA (for chemical tests) or one-way ANOVA for repeated measures (for human plasma model), followed by the *post-hoc* Tukey’s test for multiple comparisons. The correlations were evaluated by calculating Pearson correlation coefficients. Multiple regression linear models were constructed using backward elimination method and *F*-test for overall significance was performed for each model. All calculations were performed using the Satistica13Pl software for Windows (StatSoft Inc., Krakow, Poland) with *p*-values less than 0.05 regarded as significant.

## 5. Conclusions

The present study provides a new insight into the antioxidant and anti-inflammatory activity of *Cotoneaster* leaf extracts and the possibilities of their use in the prophylaxis or adjunctive therapy of oxidative stress and inflammation-related diseases. As demonstrated in chemical and biological in vitro models, the studied phenolic-rich extracts, derived from the leaves of *C. bullatus*, *C. zabelii* and *C. integerrimus*, exhibited relatively high antioxidant and anti-inflammatory effects in comparison to the positive standards. The activity of the extracts was demonstrated to arise from additive and synergistic effects of numerous polyphenolic components, among which proanthocyanidins seems to be the most important. The obtained results indicate that *Cotoneaster* extracts can be promising candidates in the development of therapeutic strategies for oxidative/nitrative stress-induced dysfunctions involved in development of various chronic diseases, especially CVD. They might also partially explain the application of *Cotoneaster* leaves in the treatment of cardiac complains reported by traditional medicine. However, further studies are required to evaluate other possible effects and mechanisms of action of the analyzed leaf extracts, e.g., their influence on the endothelium function or on the hemostatic activity of plasma and blood platelets. Finally, the real extracts effects within the cardiovascular system should be verified in vivo in animal and clinical studies.

## Figures and Tables

**Table 1:** Total phenolic content and total contents of flavonoids, phenolic acids and proanthocyanidins in the *Cotoneaster* leaf dry extracts.

Extracts ^a^	Extraction yield ^b^ (% dw)	Total Phenolics ^c^ TPC (mg GAE/g dw)	Total Flavonoids ^d^ TFC (mg/g dw)	Total Phenolic Acids ^e^ TAC (mg/g dw)	Total Proanthocyanidins	
					TPA mg CYE/g dw ^f^	TLPA mg/g dw ^g^
***C. bullatus***						
**MED**	25.60	332.94 ± 4.78 ^E^	18.12 ± 0.71 ^A^	23.37 ± 1.08 ^B^	239.62 ± 12.36 ^G^	43.25 ± 2.01 ^B^
**DEF**	0.74	546.89 ± 25.63 ^H^	66.55 ± 0.05 ^C^	30.71 ± 0.90 ^C^	33.04 ± 1.47 ^B,C^	278.66 ± 3.21 ^E^
**EAF**	4.35	650.75 ± 7.97 ^I^	62.56 ± 1.96 ^C^	37.05 ± 1.04 ^D,E^	358.22 ± 3.86 ^I^	252.47 ± 6.64 ^D^
**BF**	7.21	502.93 ± 7.98 ^G^	20.08 ± 0.27 ^A^	39.50 ± 0.42 ^E^	434.88 ± 14.57 ^J^	9.11 ± 0.09 ^A^
**WR**	14.72	101.32 ± 1.79 ^A^	nd	12.37 ± 0.67 ^A^	83.26 ± 2.04 ^D^	nd
***C. zabelii***						
**MED**	28.20	311.48 ± 8.18 ^D,E^	14.70 ± 0.05 ^A^	21.87 ± 0.29 ^B^	241.84 ± 7.27 ^G^	33.12 ± 0.40 ^B^
**DEF**	0.54	502.69 ± 25.12 ^G^	81.77 ± 1.05 ^E^	76.62 ± 0.78 ^H^	17.63 ± 0.89 ^A,B^	145.15 ± 7.03 ^C^
**EAF**	2.55	568.25 ± 10.36 ^H^	76.31 ± 1.60 ^D,E^	33.39 ± 0.54 ^C,D^	264.23 ± 13.28 ^H^	135.03 ± 2.92 ^C^
**BF**	4.61	439.07 ± 4.88 ^F^	12.66 ± 0.07 ^A^	20.93 ± 0.14 ^B^	354.06 ± 1.50 ^I^	31.42 ± 0.17 ^B^
**WR**	12.64	152.93 ± 1.45 ^B^	nd	16.12 ± 0.92 ^A^	122.10 ± 2.10 ^E^	4.17 ± 0.21 ^A^
***C. integerrimus***						
**MED**	23.33	241.25 ± 7.09 ^C^	67.71 ± 0.80 ^C,D^	71.32 ± 0.87 ^G^	128.62 ± 2.18 ^E^	nd
**DEF**	0.47	453.12 ± 3.36 ^F^	144.80 ± 2.62 ^F^	147.77 ± 1.06 ^K^	9.72 ± 0.26 ^A^	32.19 ± 0.84 ^B^
**EAF**	3.35	470.93 ± 6.19 ^F^	403.56 ± 7.48 ^G^	102.17 ± 1.79 ^I^	78.47 ± 2.26 ^D^	nd
**BF**	3.45	295.19 ± 1.34 ^D^	47.25 ± 0.31 ^B^	127.76 ± 0.06 ^J^	163.19 ± 5.51 ^F^	nd
**WR**	20.36	107.32 ± 3.54 ^A^	nd	53.35 ± 2.71 ^F^	45.60 ± 0.86 ^C^	nd

Results are the mean values ± SD (*n* = 3) calculated per dry weight (dw) of the extracts. ^a^ Extracts: MED, defatted methanol-water (7:3, *v*/*v*) extract; DEF, diethyl-ether fraction; EAF, ethyl acetate fraction; BF, *n*-butanol fraction; WR, water residue. In the columns, different superscripts (A–K) indicate significant differences in the mean values at α = 0.05; ^b^ Extraction yield calculated for dry weight of the plant material (*n* = 1); ^c^ Results expressed as GAE, gallic acid equivalents; ^d^ Results calculated as a sum of rutin and hyperoside equivalents (HPLC analysis); ^e^ Results calculated as a sum of *p*-hydroxybenzoic, chlorogenic and caffeic acids equivalents (HPLC analysis); ^f^ Results expressed as CYE, cyanidine chloride equivalents; ^g^ Results expressed as (−)-epicatechin equivalents (HPLC analysis).

**Table 2:** Antioxidant capacities of *Cotoneaster* leaf dry extract and standard antioxidants in DPPH and FRAP tests.

Extracts	DPPH ^a^		FRAP ^b^	
	EC_50_ (µg/mL)	TEAA (mmol TX/g)	(mmol Fe^2+^/g dw)	TERP (mmol TX/g)
***C. bullatus***				
**MED**	7.19 ± 0.32 ^H^	3.06 ± 0.14 ^D^	10.74 ± 0.07 ^D^	3.76 ± 0.03 ^D^
**DEF**	4.84 ± 0.03 ^D,E,F,G^	4.55 ± 0.03 ^G,H^	16.99 ± 0.78 ^I,J^	6.62 ± 0.36 ^J^
**EAF**	3.19 ± 0.10 ^B,C^	6.90 ±0.16 ^K^	17.66 ± 0.28 ^J^	6.93 ± 0.12 ^J^
**BF**	4.27 ± 0.24 ^D,E^	5.15 ± 0.29 ^H,I,J^	13.42 ± 0.15 ^E,F^	4.98 ± 0.07 ^E^
**WR**	22.41 ± 0.51 ^L^	0.98 ± 0.02 ^A,B^	3.99 ± 0.10 ^A^	1.25 ± 0.05 ^A^
***C. zabelii***				
**MED**	7.44 ± 0.03 ^H^	2.96 ± 0.01 ^D^	9.42 ± 0.13 ^C,D^	3.15 ± 0.06 ^C^
**DEF**	5.39 ± 0.09 ^F,G^	4.08 ± 0.07 ^E,F,G^	16.74 ± 0.14 ^H,I.J^	6.53 ± 0.08 ^I,J^
**EAF**	3.95 ± 0.20 ^C,D^	5.57 ± 0.28 ^J^	15.63 ± 0.16 ^G,H,I^	6.01 ± 0.07 ^G,H^
**BF**	4.78 ± 0.23 ^D,E,F^	4.60 ± 0.23 ^G,H,I^	10.81 ± 0.08 ^D^	3.80 ± 0.04 ^D^
**WR**	18.73 ± 0.33 ^K^	1.18 ± 0.02 ^A,B^	3.89 ± 0.15 ^A^	1.34 ± 0.07 ^A^
***C. integerrimus***				
**MED**	13.66 ± 0.47 ^J^	1.61 ± 0.05 ^B,C^	7.84 ± 0.25 ^B^	2.42 ± 0.11 ^B^
**DEF**	5.19 ± 0.22 ^E,F,G^	4.24 ± 0.18 ^F,G^	15.84 ± 0.12 ^G,H,I^	6.09 ± 0.05 ^H,I^
**EAF**	5.77 ± 0.06 ^G^	3.81 ± 0.04 ^E,F^	14.44 ± 0.08 ^F,G^	5.46 ± 0.04 ^F^
**BF**	10.51 ± 0.64 ^I^	2.09 ±0.13 ^C^	8.76 ± 0.33 ^B,C^	2.84 ± 0.15 ^B,C^
**WR**	27.88 ± 0.69 ^M^	0.79 ± 0.02 ^A^	3.82 ± 0.15 ^A^	1.17 ± 0.05 ^A^
***Standards***				
**QU**	1.70 ± 0.11 ^A^	9.00 ± 0.60 ^L^	31.20 ± 0.98 ^L^	11.88 ± 0.01 ^M^
**ECA**	2.35 ± 0.18 ^A,B^	6.98 ± 0.18 ^K^	35.79 ± 0.93 ^M^	8.22 ± 0.26 ^L^
**CHA**	4.60 ± 0.07 ^D,E,F^	3.52 ± 0.07 ^D,E^	25.68 ± 0.51 ^K^	5.59 ± 0.15 ^F,G^
**BHA**	2.90 ± 0.15 ^B^	5.20 ± 0.26 ^I,J^	16.14 ± 0.77 ^H,I^	7.73 ± 0.01 ^K^
**TX**	4.05 ± 0.10 ^C,D^	-	12.69 ± 0.42 ^E^	-

Results are the mean values ± SD (*n* = 3) calculated per dry weight (dw) of the extracts. Extracts: MED, methanol-water (7:3, *v*/*v*) extract; DEF, diethyl-ether fraction; EAF, ethyl acetate fraction; BF, *n*-butanol fraction; WR, water residue. Standards: QU, quercetin, ECA, (−)-epicatechin; CHA, chlorogenic acid; BHA, butylated hydroxyanisole; TX, Trolox^®^. In the columns, different superscripts (A–M) indicate significant differences in the mean values at α = 0.05. ^a^ Radical-scavenging efficiency expressed as effective concentration (EC_50_, amount of the dry extracts or standards needed to decrease the initial DPPH concentration by 50%) or Trolox^®^ equivalent antioxidant activity (TEAA). ^b^ Ferric reducing antioxidant power expressed in milimoles of ferrous ions/g of dry extracts or as Trolox^®^ equivalent reducing power (TERP).

**Table 3:** Inhibitory effects of *Cotoneaster* leaf extract and standards on lipoxygenase (LOX) and hyaluronidase (HYAL).

Extracts	LOX		HYAL	
	IC_50_^a^ (µg/mL)	IC_50_^b^ (µg/U)	IC_50_^a^ (µg/mL)	IC_50_^b^ (µg/U)
***C. bullatus***				
**MED**	185.77 ± 9.08 ^E,F^	6.85 ± 0.33 ^E,F^	8.09 ± 0.67 ^B^	7.09 ± 0.59 ^B^
**DEF**	217.01 ± 2.60 ^F^	8.00 ± 0.10 ^F^	8.35 ± 0.53 ^B,C^	7.32 ± 0.46 ^B,C^
**EAF**	129.29 ± 7.38 ^C,D^	4.77 ± 0.27 ^C,D^	19.09 ± 0.62 ^G^	16.75 ± 0.55 ^G^
**BF**	95.19 ± 4.31 ^A,B^	3.51 ± 0.16 ^A,B^	2.81 ± 0.13 ^A^	2.46 ± 0.11 ^A^
**WR**	595.31 ± 27.09 ^I^	21.96 ± 1.00 ^I^	27.40 ± 1.14 ^I^	24.04 ± 1.00 ^I^
***C. zabelii***				
**MED**	217.80 ± 12.56 ^F^	8.03 ± 0.46 ^F^	7.92 ± 0.25 ^B^	6.94 ± 0.22 ^B^
**DEF**	202.21 ± 8.93 ^F^	7.46 ± 0.33 ^F^	7.82 ± 0.16 ^B^	6.86 ± 0.14 ^B^
**EAF**	136.39 ± 9.27 ^C,D^	5.03 ± 0.34 ^C,D^	15.53 ± 0.40 ^E,F^	13.63 ± 0.35 ^E,F^
**BF**	130.99 ± 3.78 ^C,D^	4.83 ± 0.14 ^C,D^	6.08 ± 0.19 ^B^	5.33 ± 0.17 ^B^
**WR**	427.68 ± 12.47 ^H^	15.78 ± 0.46 ^H^	12.70 ± 0.66 ^D,E^	11.14 ± 0.58 ^D,E^
***C. integerrimus***				
**MED**	283.55 ± 14.12 ^G^	10.46 ± 0.52 ^G^	24.00 ± 0.93 ^H^	21.05 ± 0.82 ^H^
**DEF**	136.11 ± 8.10 ^C,D^	5.02 ± 0.30 ^C,D^	11.21 ± 0.07 ^C,D^	9.83 ± 0.06 ^C,D^
**EAF**	159.21 ± 3.97 ^D,E^	5.87 ± 0.15 ^D,E^	30.59 ± 2.18 ^J^	26.83 ± 1.91 ^J^
**BF**	199.42 ± 6.41 ^F^	7.36 ± 0.24 ^F^	11.09 ± 0.29 ^C,D^	9.73 ± 0.26 ^C,D^
**WR**	684.84 ± 18.86 ^J^	25.26 ± 0.70 ^J^	34.39 ± 0.61 ^K^	30.16 ± 0.53 ^K^
***Standards***				
**QU**	57.83 ± 1.47 ^A^	2.15 ± 0.04 ^A^	15.84 ± 0.87 ^F^	13.90 ± 0.76 ^F^
**ECA**	90.59 ± 0.50 ^A,B^	3.34 ± 0.02 ^A,B^	14.27 ± 0.20 ^D,E,F^	12.52 ± 0.18 ^D,E,F^
**CHA**	114.25 ± 2.87 ^B,C^	4.21 ± 0.11 ^B,C^	16.53 ±0.34 ^F,G^	14.50 ± 0.30 ^F,G^
**IND**	62.95 ± 2.19 ^A^	2.32 ± 0.08 ^A^	8.61 ± 0.22 ^B,C^	7.77 ± 019 ^B,C^

Results are the mean values ± SD (*n* = 3) calculated per dry weight (dw) of the extracts. Extracts: MED, methanol-water (7:3, *v*/*v*) extract; DEF, diethyl-ether fraction; EAF, ethyl acetate fraction; BF, *n*-butanol fraction; WR, water residue. Standards: QU, quercetin, ECA, (−)-epicatechin; CHA, chlorogenic acid; IND, indomethacin. In the columns, different superscripts (A–K) indicate significant differences in the mean values at α = 0.05. The inhibitory activity toward lipoxygenase (LOX) and hyaluronidase (HYAL) was expressed as IC_50_ (amount of analyte needed for 50% inhibition of enzyme activity): ^a^ in µg of the dry extracts or standards/mL of the enzyme solution; ^b^ in µg of the extracts/enzyme unit (U).

## Supplementary Materials

The following are available online. Table S1: UHPLC-PDA-ESI-MS^3^ data of polyphenols identified in the fractionated dry extracts of *Cotoneaster* leaves; Figure S1: The UHPLC-UV chromatograms of the leaf extracts of *C. bullatus*, *C. zabelii* and *C. integerrimus* at 280 nm, Table S2: Correlation coefficients (*r*) of the linear relationships between antioxidant activity parameters and the contents of particular groups of phenolics in the investigated *Cotoneaster* extracts; Table S3: Statistically significant multiple regression models for activity parameters based on the content of particular groups of phenolics in the *Cotoneaster* extracts.
