# Supplementary material for: Polyphenol-Rich Extracts from Cotoneaster Leaves Inhibit Pro-Inflammatory Enzymes and Protect Human Plasma Components against Oxidative Stress In Vitro
Source: Molecules. 2018 Sep 26;23(10):2472. doi: 10.3390/molecules23102472 (PMC6222437; doi:10.3390/molecules23102472)
Supplement: Supplementary file 1 [file molecules-23-02472-s001.pdf]

Molecules **2018**, *23*, 2472; doi: 10.3390/molecules23102472

Supplementary Materials:

Polyphenol-Rich Extracts from *Cotoneaster* Leaves Inhibit Pro-Inflammatory Enzymes and Protect Human Plasma Components Against Oxidative Stress in vitro

Agnieszka Kicel, Joanna Kolodziejczyk-Czepas, Aleksandra Owczarek, Anna Marchelak, Malgorzata Sopinska, Pawel Ciszewski, Pawel Nowak, Monika A. Olszewska

Results

Qualitative UHPLC-PDA-ESI-MS³ profiling of Cotoneaster leaf phenolics

The qualitative UHPLC-PDA-ESI-MS^3 survey on the *Cotoneaster* leaf extracts revealed the presence of over forty phenolic compounds (Figure 1, Table S1), that were divided into three main groups such as phenolic acids and related quinic acid pseudodepsides, flavonoids, and flavan-3-ol derivatives including procyanidins.

In the group of quinic acid mono- and diesters containing in their structure caffeic and *p*-coumaroyl moieties (**3**, **4**, **6**, **7**, **9**, **12**, **13**, **17**, **21**, **42** and **46**), compounds **3**, **6**, **9**, **13**, **42** and **46** were identified for the first time in the *Cotoneaster* species. Compound **6** was proposed to be a caffeic acid derivative, because its [M–H]$^-$ ion at *m*/*z* 451 yielded the MS^2 peak at *m*/*z* 179, typical of caffeic acid moiety. Compounds **9**, **13**, **42** and **46**, eluting after chlorogenic acid (**7**) displayed the parent [M–H]$^-$ ions at *m*/*z* 515, suggesting that all four analytes were dicaffeoylquinic acids. In the case of compounds **9** and **13**, despite the occurrence of some MS^2 fragments characteristic for dicaffeoylquinic acids, their MS fragmentation pattern was not characteristic enough to assign to any particular structure [1]. On the other hand, the MS^2 base peak of **42** and **46** at *m*/*z* 353, produced by the loss of a caffeoyl moiety [M–H–caffeoyl]$^-$, and its subsequent fragmentation evaluated by the hierarchical key [1], allowed for identification of compounds **45** and **46** as 3,5- and 4,5-*O*-dicaffeoylquinic acids, respectively. The peak **3,** also displaying the parent [M–H]$^-$ ion at *m*/*z* 515, was eluted much earlier than those of dicaffeoylquinic acids (**9**, **13**, **42**, **46**) and chlorogenic acid (**7**). In addition to the fragments characteristic of monocaffeoylquinic acids, the MS^2 spectrum of this compound yielded fragments at *m*/*z* 353 ([M-H-162]$^-$, loss of a hexose residue) and 341 ([M–H–174]$^-$, loss of a quinic acid moiety). This fragmentation pattern pointed to a glycosylated monocaffeoylquinic acid, which might correspond to 1 or 5-*O*-caffeoylquinic acid hexoside [1]. The fact, that 5-*O*-caffeoylquinic acid (chlorogenic acid) was the major isomer in the tested extracts, permitted tentative identification of compound **3** as 5-*O*-caffeoylquinic acid hexoside.

Compounds **1**, **2** and **10**, detected only in DEF fractions were identified by comparison with reference standards and literature data [2] as protocatechuic, *p*-hydroxybenzoic and caffeic acids.

Among flavan-3-ols and procyanidins, seventeen individuals were found (**5**, **8**, **11**, **14-16**, **18-20**, **22-25**, **27**, **28**, **35** and **36**), among which (−)-epicatechin (**18**), procyanidins B-2 (**15**) and C-1 (**23**) clearly dominated, especially in the DEF and EAF fractions. In this group, seven analytes displaying parent ([M–H]$^-$ ions at *m*/*z* 577 (**5**, **11**, **14**, **19**, **22**, **24**) or at *m*/*z* 1153 (**20**) and the relevant MS^2 and MS^3 fragments corresponding to B-type procyanidin di- and tetramers, respectively [3] were identified in the analyzed *Cotoneaster* species for the first time. Additionally, compound **36** was proposed to be proanthocyanidin derivative, because its parent [M–H]$^-$ ion at *m*/*z* 483 yielded the MS^2 fragment at *m*/*z* 289, characteristic for epicatechin.

Of the compounds classified as flavonoids (**26**, **30-34**, **38-41**, **43-45** and **47**), all analytes were found to be quercetin or kaempferol mono- and diglycosides. Compounds **26**, **32**, **33**, **34**, **41** and **47** were identified with authentic standards as quercetin 3-*O*-*β*-(2''-*O*-*β*-xylosyl)-galactoside, hyperoside, rutin, isoquercitrin, quercitrin, and quercetin, respectively. Among three compounds identified in the

Cotoneaster genus for the first time, **38** with the parent $[M–H]^-$ ion at *m/z* 433 was suggested to be quercetin pentoside, while compound **39** with the $[M–H]^-$ ion at *m/z* 447 was classified as kaempferol hexoside. Compound **45** with $[M–H]^-$ ion at *m/z* 593 was tentatively characterized as quercetin dirhamnoside.

References

1. Clifford, M.N.; Knight, S.; Kuhnert, N. Discriminating between the six isomers of dicaffeoylquinic acid by LC-MSn. *J. Agric. Food Chem.*, **2005**, *53*, 3821–3832.
2. Su, S.; Cui, W.; Zhou, W.; Duan, J. A.; Shang, E.; Tang, Y. Chemical fingerprinting and quantitative constituent analysis of Siwu decoction categorized formulae by UPLC-QTOF/MS/MS and HPLC-DAD. *Chin. Med.*, **2013**, *8*, 1-15.
3. Karar, M. G. E.; Kuhnert, N. UPLC-ESI-Q-TOF-MS/MS characterization of phenolics from *Crataegus monogyna* and *Crataegus laevigata* (Hawthorn) leaves, fruits and their herbal derived drops (Crataegutt Tropfen). *J. Chem. Biol. Ther.* **2015**, 1, 1-23.

Molecules **2018**, *23*, 2472; doi: 10.3390/molecules23102472

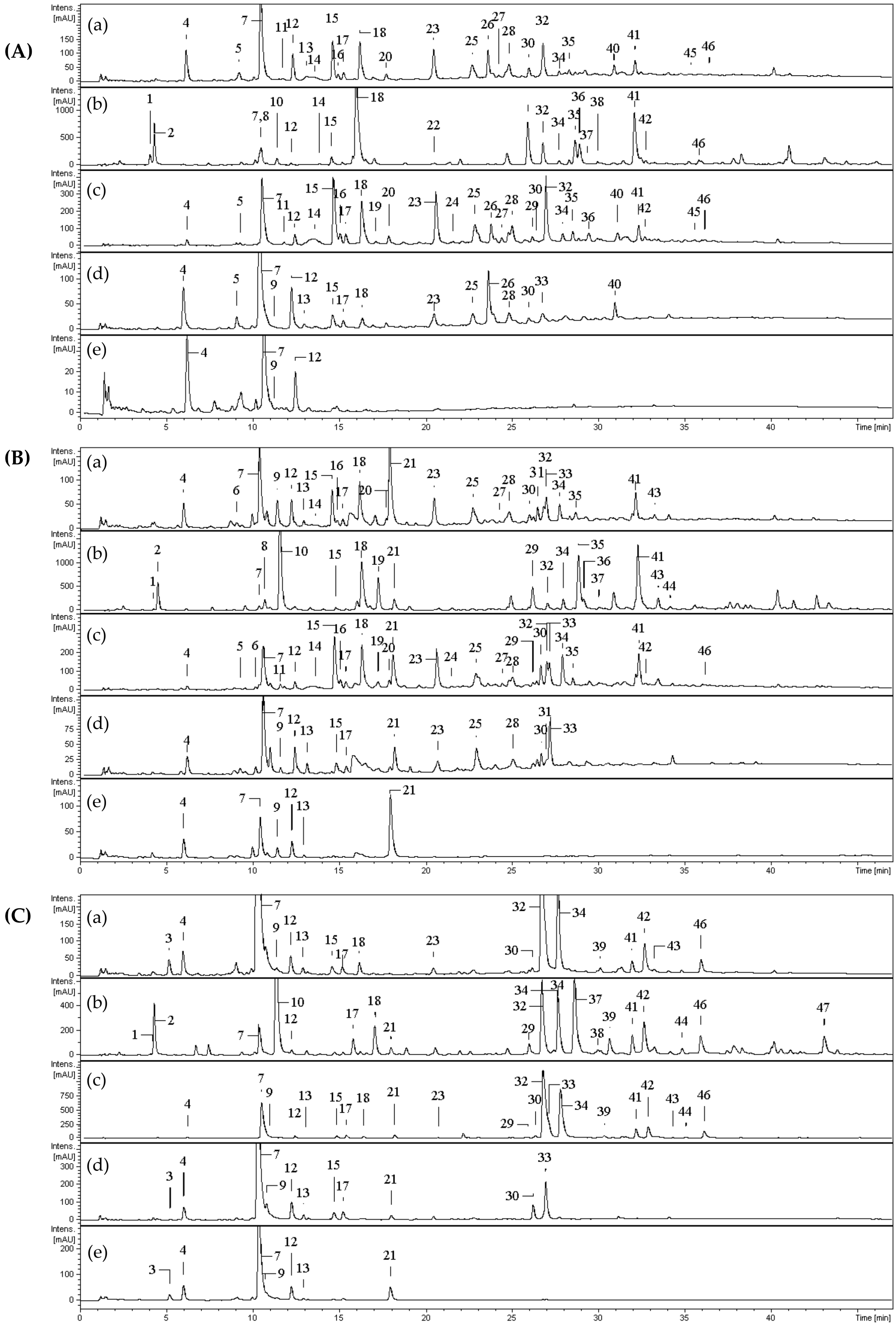

Figure S1. The UHPLC-UV chromatograms of the leaf extracts of *C. bullatus* (A), *C. zabelii* (B) and *C. integerrimus* (C) at 280 nm. Extracts: (a) MED, defatted methanol-water (7:3, *v*/*v*) extract; (B) DEF, diethyl-ether fraction; (c) EAF, ethyl acetate fraction; (d) BF, *n*-butanol fraction; (e) WR, water residue.

Table S1. UHPLC-PDA-ESI-MS^3 data of polyphenols identified in the fractionated dry extracts of *Cotoneaster* leaves.

Peak	Analyte	R_t [c] (min)	UV λ_{max} [d] (nm)	Formula	$[M-H]^-$ [e] (*m*/*z*)	Fragmentary ions (% relative abundance)	Extracts [f]				
							MED	DEF	EAF	BF	WR
1	protocatechuic acid [a, b]	4.4	260, 293	$C_7H_6O_4$	153	MS^2: 153(100)		B, Z, I			
2	*p*-hydroxybenzoic acid [a, b]	4.7	254	$C_7H_6O_3$	137	MS^2: 93(40)		B, Z, I			
3	caffeoylquinic acid hexoside [b]	5.4	325	$C_{25}H_{24}O_{12}$	515	MS^2: 379(69),353(29), 341 (20), 191(23) MS^3[353]:191(84)	I			I	I
4	3-*O*-caffeoylquinic acid (neochlorogenic acid) [a]	6.1	325	$C_{16}H_{18}O_9$	353	MS^2: 191(100), 179(40), 135(5)	B, Z, I		B, Z, I	B, Z, I	B, Z, I
5	procyanidin dimer B-type [b]	9.0	280	$C_{30}H_{26}O_{12}$	577	MS^2: 451(33), 425(100), 407(65), 289(26) MS^3[425]: 407(88), 273(10)	B		B, Z	B	
6	caffeic acid derivative [b]	9.7	325		451	MS^2: 405 (100), 179 (7)	Z		Z		
7	5-*O*-caffeoylquinic acid (chlorogenic acid) [a]	10.4	325	$C_{16}H_{18}O_9$	353	MS^2: 191(96), 179(3)	B, Z, I	B, Z, I	B, Z, I	B, Z, I	B, Z, I
8	(+)-catechin [a]	10.4	280	$C_{15}H_{14}O_6$	289	MS^2: 245 (100), 205 (41)		B, Z			
9	dicaffeoylquinic acid isomer [b]	11.1	325	$C_{25}H_{24}O_{12}$	515	MS^2: 395(22), 379(100), 285(8)	Z, I		I	B, Z, I	B, Z, I
10	caffeic acid [a, b]	11.6	325	$C_9H_8O_4$	179	MS^2: 135(87)		B, Z, I			
11	procyanidin dimer B-type [b]	11.7	280	$C_{30}H_{26}O_{12}$	577	MS^2: 451(17), 425(100), 407 (64), 289(15) MS^3[425]: 407(100), 273(11)	B		B, Z		
12	4-*O*-caffeoylquinic acid [a] (cryptochlorogenic acid)	12.3	325	$C_{16}H_{18}O_9$	353	MS^2: 191(28), 179(61), 173 (100)	B, Z, I	B, I	B, Z, I	B, Z, I	B, Z, I
13	dicaffeoylquinic acid isomer [b]	12.8	325	$C_{25}H_{24}O_{12}$	515	MS^2: 395(16), 379(100), 285(67)	B, Z, I		I	B, Z, I	Z, I
14	procyanidin dimer B-type [b]	13.5	280	$C_{30}H_{26}O_{12}$	577	MS^2: 451(44), 425(100), 407(64), 289(15) MS^3[425]: 407(100), 273(6)	B, Z	B	B, Z		
15	procyanidin B2 [a]	14.6	280	$C_{30}H_{26}O_{12}$	577	MS^2: 451(27), 425(100), 407(41), 289(8) MS^3[425]: 407(100), 273(4)	B, Z, I	B, Z	B, Z, I	B, Z, I	
16	procyanidin trimer B-type	15.0	280	$C_{45}H_{38}O_{18}$	865	MS^2: 739(93), 713(58), 695(87), 577(20) MS^3[713]: 695(76), 425(73), 407(100)	B, Z		B, Z		
17	5-*p*-coumaroylquinic acid	15.3	310	$C_{16}H_{18}O_8$	337	MS^2: 191(100), 163(5)	B, Z, I	I	B, Z, I	B, Z, I	
18	(-)-epicatechin [a]	16.1	280	$C_{15}H_{14}O_6$	289	MS^2: 245(100), 205(22), 179(13), 137(5)	B, Z, I	B, Z, I	B, Z, I	B	
19	procyanidin dimer B-type [b]	17.2	280	$C_{30}H_{26}O_{12}$	577	MS^2: 451(15), 425(100), 407(19), 289(24)		Z	B, Z		
20	procyanidin tetramer B-type [b]	17.7	280	$C_{60}H_{50}O_{24}$	1153	MS^2: 1027(47), 863(72), 739(14), 501(60), 491(81), 289(100)	B, Z		B, Z		
21	caffeic acid derivative	18.2	290, 328		613	MS^2: 457(6), 339(14), 295(100), 179(16)	Z	Z, I	Z, I	Z, I	Z, I
22	procyanidin dimer B-type [b]	20.3	280	$C_{30}H_{26}O_{12}$	577	MS^2: 451(17), 425(100), 407(59), 289(20) MS^3[425]: 407(100), 273(12)		B			
23	procyanidin C1 [a]	20.4	280	$C_{45}H_{38}O_{18}$	865	MS^2: 713(51), 695(100), 577(26) MS^3[713]: 695(100), 425(32), 407(36)	B, Z, I		B, Z, I	B, Z	

24	procyanidin dimer B-type [b]	21.4	280	$C_{30}H_{26}O_{12}$	577	MS²: 425(91), 407(72), 289(100)<br>MS³[425]: 407(100), 273(4)			B, Z	
25	procyanidin tetramer B-type	22.9	280	$C_{60}H_{50}O_{24}$	1153	MS²: 1027(25), 863(69), 739(3), 501(51), 491(39), 289(100)	B, Z		B, Z	B, Z
26	quercetin 3-*O*-ß-(2′′-*O*-ß-xylosyl)galactoside [a]	23.6	268, 355	$C_{26}H_{28}O_{16}$	595	MS²: 463(9), 445(15), 301(100)	B		B	B
27	procyanidin tetramer B-type	24.3	280	$C_{60}H_{50}O_{24}$	1153	MS²: 1027(17), 863(94), 739(14), 501(100), 491(71), 289(97)	B, Z		B, Z	
28	procyanidin dimer hexoside	24.9	280	$C_{36}H_{36}O_{17}$	739	MS²: 587(100), 577 (12), 451(18), 289(32)	B, Z		B, Z	B, Z
29	unidentified compound	26.1	280		451	MS²: 341 (100), 217 (4)		B, Z, I	B, I	
30	quercetin rhamnoside-hexoside	26.4	255, 355	$C_{27}H_{30}O_{16}$	609	MS²: 447(7), 343(12), 301(100)<br>MS³[447]: 301(100)	B, Z, I		B, Z, I	B, Z, I
31	quercetin dirhamnoside	26.6	255, 350	$C_{27}H_{30}O_{15}$	593	MS²: 447(100), 301(45)<br>MS³[447]: 301(100)			Z	Z
32	hyperoside (quercetin 3-*O*-ß-galactoside) [a]	26.9	255, 353	$C_{21}H_{20}O_{12}$	463	MS²: 301(100)	B, Z, I	B, Z, I	B, Z, I	
33	rutin (quercetin 3-*O*-ß-(6′′-*O*-α-rhamnosyl)-glucoside) [a]	27.3	265, 350	$C_{27}H_{30}O_{16}$	609	MS²: 463(1), 343(6), 301(100)	Z		Z, I	B, Z, I
34	isoquercitrin (quercetin-*O*-ß-glucoside) [a]	27.9	275, 350	$C_{21}H_{20}O_{12}$	463	MS²: 301(100)	B, Z, I	B, Z, I	B, Z, I	
35	procyanidin dimer B-type	28.5	280	$C_{30}H_{26}O_{12}$	577	MS²: 425(100), 407(51), 289(16)	B, Z	B, Z	B, Z	
36	proanthocyanidin derivative [b]	28.8	280		483	MS²: 451(61), 341(42), 289(100), 245(4)		B, Z	B	
37	unidentified compound	29.0	280		451	MS²: 341(91), 299(100), 189(22), 177(23)		B, Z, I		
38	quercetin pentoside [b]	30.2	275, 350	$C_{20}H_{18}O_{11}$	433	MS²: 301(100), 179(4)		B, I		
39	kaempferol hexoside [b]	30.5	265, 350	$C_{21}H_{20}O_{11}$	447	MS²: 285(100)	I	I	I	
40	quercetin rhamnoside-hexoside	31.0	265, 355	$C_{27}H_{30}O_{16}$	609	MS²: 447(9), 301(100)	B		B	B
41	quercitrin (quercetin 3-*O*-ß-rhamnoside) [a]	32.2	275, 350	$C_{21}H_{20}O_{11}$	447	MS²: 301(100)	B, Z, I	B, Z, I	B, Z, I	
42	3,5-*O*-dicaffeoylquinic acid [b]	32.9	325	$C_{25}H_{24}O_{12}$	515	MS²: 447(19), 379(57), 353(100)<br>MS³[353]: 191(100), 179(35)	I	B, I	B, Z, I	
43	quercetin hexoside derivative	33.3	265, 355	$C_{23}H_{22}O_{13}$	505	MS²: 463(24), 337(26), 301(100)	Z, I	Z	I	
44	quercetin hexoside derivative	34.7	265, 355	$C_{23}H_{22}O_{13}$	505	MS²: 463(31), 373(4), 301(100)		Z, I	I	
45	quercetin dirhamnoside [b]	35.3	365, 355	$C_{27}H_{30}O_{15}$	593	MS²: 447(11), 301(100)	B		B	
46	4,5-*O*-dicaffeoylquinic acid isomer [b]	36.3	325	$C_{25}H_{24}O_{12}$	515	MS²: 379(90), 353(100), 299(8), 203(14)<br>MS³[353]: 191(37), 179(69), 173(100	I, B	B, I	B, Z, I	
47	quercetin [a, b]	43.2	256, 365	$C_{15}H_{10}O_7$	301	MS²: 273(11), 179(100), 151(66), 107(5)		Z, I		

[a] Analytes identified with authentic standards. [b] Compounds identified in the *Cotoneaster* extracts for the first time. [c] R_t, retention time. [d] UVmax, absorbance maxima in PDA spectra. [e] $[M-H]^-$, pseudomolecular ion in MS spectra recorded in a negative mode. [f] MED, methanol-water (7:3, v/v) extract; DEF, diethyl-ether fraction; EAF, ethyl acetate fraction; BF, *n*-butanol fraction; WR, water residue; **B**, *C. bullatus*; **Z**, *C. zabelii* and **I**, *C. integerrimus*.

Table S2. Correlation coefficients (*r*) of the linear relationships between antioxidant activity parameters and the content of particular groups of phenolics in the investigated *Cotoneaster* extracts.

Group of phenolics	Chemical model		Human plasma model					Enzyme inhibition	
	DPPH (mmol TX/g)	FRAP (mmol TX/g)	3NT (nmol/mg)	LOOH (nmol/mg)	TBARS (μmol/mL)	DPPH (μM TX)	FRAP (mM Fe^{2+})	HYAL	LOX
TPC (mg GAE/g dw)	**0.9706*****	**0.9622*****	–0.6541	0.0948	–0.6154	**0.7823***	0.6009	–0.4228	**–0.8403*****
TFC (mg/g dw)	0.2386	0.4378	–0.0211	–0.1395	0.5604	0.2394	0.2103	0.3407	–0.3416
TAC (mg/g dw)	0.0012	0.2457	0.4858	–0.1351	0.6621	–0.2518	–0.1496	0.1116	–0.2599
TPA (mg CYE/g dw)	0.5088	0.1160	0.2312	**0.7432***	–0.1747	–0.2562	–0.2919	–0.4071	–0.4707
TLPA (mg/g dw)	**0.6645****	**0.7240****	**–0.8784****	–0.3891	**–0.8675****	**0.7263***	0.6080	–0.2326	–0.3326

Activity and quantitative parameters according to Tables 1, 2 and Figures 2, 3. Asterisks mean statistical significance of the estimated linear relationships (*$p < 0.05$, **$p < 0.01$, ***$p < 0.001$).

Table S3. Statistically significant multiple regression models for activity parameters based on the content of particular groups of phenolics in the *Cotoneaster* extracts.

Activity parameter	Statistically significant coefficients (*p*)					Parameters of the model		
	Y-intercept	TFC	TAC	TPA	TLPA	adj. R^2	*F*-value	*p*-value
DPPH (mmol TX/g)[a]	-	0.0055 (0.0290)	0.0114 (0.0187)	0.0092 (0.0000)	0.0136 (0.0000)	0.9683	115.75	0.0000
FRAP (mmol TX/g)[a]	-	0.0073 (0.0329)	0.0228 (0.0019)	0.0068 (0.0005)	0.0185 (0.0000)	0.9591	89.09	0.0000
LOX (nmol/mL)[a]	608.9 (0.0000)	-	–2.705 (0.0058)	–0.9297 (0.0026)	–0.8006 (0.0301)	0.5806	7.46	0.0053
DPPH (μM TX)[b]	0.4536 (0.0000)	-	-	-	0.0002 (0.0267)	0.4601	7.817	0.0267
TBARS (μmol/mL)[b]	0.0442 (0.0000)	-	-	-	–0.00002 (0.0024)	0.7172	21.29	0.0024
3-NT (nmol/mg)[b]	2.3414 (0.0000)	-	-	-	–0.0023 (0.0018)	0.7390	23.66	0.0018
LOOH (nmol/mg)[b]	0.6243 (0.0008)	-	-	0.0013 (0.0217)	-	0.4884	8.64	0.0217

Activity and quantitative parameters according to Tables 1, 2 and Figures 2, 3; the activity parameters from [a] the chemical and [b] the human plasma tests;
adj. R^2, R^2 adjusted for number of predictors in the model; *F*-value, *F*-test for overall significance
